# Poly[[hepta-μ_2_-aqua-bis­(μ_2_-pyrazine-2-carboxyl­ato)dibarium] bis­(pyrazine-2-carboxyl­ate)]

**DOI:** 10.1107/S1600536811003023

**Published:** 2011-01-29

**Authors:** Qi Shuai, Ke-Lian Ding, Fan Hu, Ping Yu

**Affiliations:** a College of Science, Northwest A&F University, Yangling 712100, Shanxi Province, People’s Republic of China; b Students Service, Northwest A&F University, Yangling 712100, Shanxi Province, People’s Republic of China

## Abstract

In the layered title coordination polymer, {[Ba_2_(C_5_H_3_N_2_O_2_)_2_(H_2_O)_7_](C_5_H_3_N_2_O_2_)_2_}_*n*_, the coordination geometries around the two independent Ba^II^ ions can be described as bicapped square-anti­prismatic [BaNO_9_] arrangements. A two-dimensional polymeric framework with (6,3) topology can be observed in the *ac* plane, the nodes being provided by Ba^II^ ions and the connectors being N and O atoms belonging to pyrazine-2-carboxyl­ate ligands and O atoms of bridging water mol­ecules. Non-coordinating pyrazine-2-carboxyl­ate ions are located between the polymeric layers in the crystal and are interconnected through extensive O—H⋯N,O hydrogen bonding.

## Related literature

For Ca^II^ and Sr^II^ complexes with pyrazine-2-carboxyl­ate as ligand, see: Ptasiewicz-Bak *et al.* (1998 ▶). For different modes of coordination for pyrazine-2-carboxyl­ate in polymers, see: Huang *et al.* (2003 ▶); Yin *et al.* (2006 ▶).
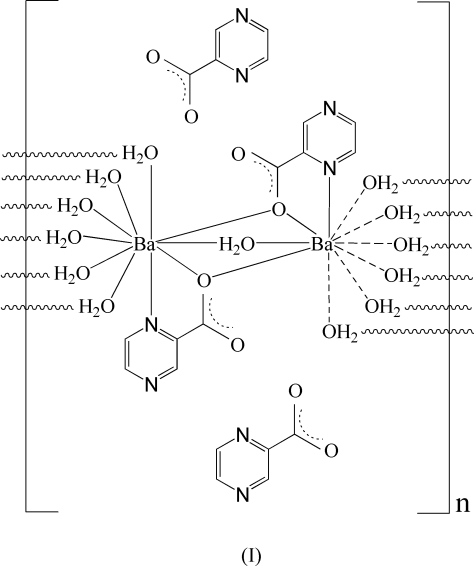


## Experimental

### 

#### Crystal data

[Ba_2_(C_5_H_3_N_2_O_2_)_2_(H_2_O)_7_](C_5_H_3_N_2_O_2_)_2_
*M* *_r_* = 893.17
Monoclinic, 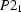

*a* = 7.5652 (10) Å
*b* = 29.263 (3) Å
*c* = 7.6067 (10) Å
β = 118.741 (2)°
*V* = 1476.5 (3) Å^3^
*Z* = 2
Mo *K*α radiation
μ = 2.74 mm^−1^
*T* = 298 K
0.49 × 0.34 × 0.16 mm

#### Data collection

Bruker SMART APEX CCD area-detector diffractometer
Absorption correction: multi-scan (*SADABS*; Bruker, 2002 ▶) *T*_min_ = 0.348, *T*_max_ = 0.669
7342 measured reflections
4734 independent reflections
4502 reflections with *I* > 2σ(*I*)
*R*_int_ = 0.036

#### Refinement

*R*[*F* ^2^ > 2σ(*F* ^2^)] = 0.046
*wR*(*F* ^2^) = 0.121
*S* = 1.02
4734 reflections
406 parameters
1 restraint
H-atom parameters constrained
Δρ_max_ = 1.84 e Å^−3^
Δρ_min_ = −2.62 e Å^−3^
Absolute structure: Flack (1983 ▶), 2096 Friedel pairs
Flack parameter: 0.04 (4)

### 

Data collection: *SMART* (Bruker, 2002 ▶); cell refinement: *SAINT* (Bruker, 2002 ▶); data reduction: *SAINT*; program(s) used to solve structure: *SHELXS97* (Sheldrick, 2008 ▶); program(s) used to refine structure: *SHELXL97* (Sheldrick, 2008 ▶); molecular graphics: *SHELXTL* (Sheldrick, 2008 ▶); software used to prepare material for publication: *SHELXTL*.

## Supplementary Material

Crystal structure: contains datablocks global, I. DOI: 10.1107/S1600536811003023/bh2331sup1.cif

Structure factors: contains datablocks I. DOI: 10.1107/S1600536811003023/bh2331Isup2.hkl

Additional supplementary materials:  crystallographic information; 3D view; checkCIF report

## Figures and Tables

**Table 1:** Hydrogen-bond geometry (Å, °)

*D*—H⋯*A*	*D*—H	H⋯*A*	*D*⋯*A*	*D*—H⋯*A*
O10—H10*A*⋯O7	0.85	2.23	3.000 (13)	152
O10—H10*B*⋯O5	0.85	1.92	2.769 (11)	177
O11—H11*A*⋯O4	0.85	1.88	2.669 (12)	153
O11—H11*B*⋯O5	0.85	2.04	2.861 (12)	163
O11—H11*B*⋯N5	0.85	2.62	3.191 (14)	126
O13—H13*A*⋯N5	0.85	2.14	2.983 (14)	175
O14—H14*A*⋯O8	0.85	1.87	2.685 (12)	160
O9—H9*A*⋯O7^i^	0.85	1.88	2.677 (12)	155
O9—H9*B*⋯O5^i^	0.85	1.89	2.689 (12)	156
O13—H13*B*⋯O6^i^	0.85	1.93	2.729 (12)	157
O15—H15*A*⋯O7^i^	0.85	2.04	2.860 (12)	161
O15—H15*A*⋯N7^i^	0.85	2.62	3.208 (14)	127
O12—H12*A*⋯O7^ii^	0.85	1.96	2.811 (12)	179
O12—H12*B*⋯O5^ii^	0.85	2.26	3.018 (12)	149
O14—H14*B*⋯N7^ii^	0.85	2.18	3.028 (13)	174
O15—H15*B*⋯O2^iii^	0.85	1.90	2.676 (11)	152

Symmetry codes: (i) 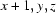; (ii) 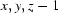; (iii) 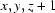.

**Fig. 1.**
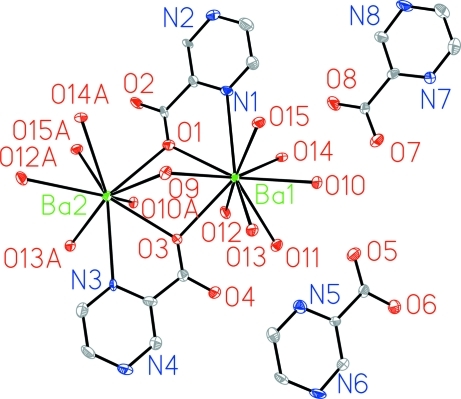
Coordination environment of BaII ions in the title complex. Non-hydrogen atoms are shown as 30% probability ellipsoids. Hydrogen atoms are omitted for clarity.

**Fig. 2.**
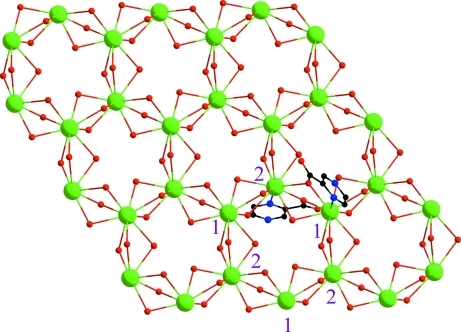
View of one two-dimensional layer structure along b axis in the title complex. Hydrogen atoms are omitted for clarity. Some redundant atoms are omitted for clarity.

## supplementary crystallographic information

### Comment

Ptasiewicz-Bak *et al.* obtained monomeric complexes of calcium and strontium [*M*(C_5_H_3_N_2_O_2_)_2_(H_2_O)_4_] (*M* = Ca^II^ or Sr^II^, Ptasiewicz-Bak *et al.*, 1998), based on 2-pyrazinecarboxylate ligand, which are isostructural.

Here, we report a complex, (I), assembled by alkaline earth metal Ba^II^ ion with 2-pyrazinecarboxylate as ligand. Different from complexes of calcium and strontium, the formula for the title complex is [Ba_2_(C_5_H_3_N_2_O_2_)_2_(H_2_O)_7_] (C_5_H_3_N_2_O_2_)_2_. X-ray single-crystal diffraction analysis indicates the presence of two independent Ba^II^ ions, two coordinated pyrazine-2-carboxylate ions, seven coordinated water molecules and two isolated pyrazine-2-carboxylate ions in the asymmetric unit. Only one independent Ca^II^ or Sr^II^ ions are found in the complexes reported by Ptasiewicz-Bak *et al.*

In the title complex, the coordination geometries (Fig. 1) around Ba1 and Ba2 centres could be described as bicapped square-antiprismatic [BaNO_9_] arrangements with coordination number of 10, where one N and two O atoms come from 2-pyrazinecarboxylate ligands, the rest, seven O atoms, being from seven coordinated water molecules. There are two kinds of pyrizine-2-carboxylate coordination modes, which have been reported previously (Yin *et al.*, 2006; Huang *et al.*, 2003). In (I), only one kind of coordination mode, µ_2_ bridging mode, is observed. All the water molecules are coordinated and act as µ_2_ bridging ligands. In this case, every six Ba^II^ ions form metal hexameric rings which share common edges, to construct two-dimensional, infinite networks with (6,3) topology (Fig. 2) parallel to the *ac* plane. Within the (6,3) topology layer, the nodes are provided by Ba^II^ and the connectors are N and O atoms which come from 2-pyrazinecarboxylate ions, and O atoms of water molecules. Non coordinating pyrazine-2-carboxylate ions are placed between polymeric layers in the crystal.

### Experimental

A mixture of barium chloride dihydrate (0.0244 g, 0.1 mmol), sodium hydroxide (0.0160 g, 0.4 mmol), 2-pyrazinecarboxylic acid (0.0496 g, 0.4 mmol), and H_2_O (3 ml) was placed in a Parr Teflon-lined stainless steel vessel (25 ml). The vessel was sealed and heated to 443.15 K for 6 days. Then, the vessel was cooled to 373.15 K at a rate of 5 K.h^-1^ and slowly cooled to room temperature. Colourless, rectangular single crystals suitable for X-ray diffraction were obtained.

### Refinement

All H atoms were placed in geometrically idealized positions and constrained to ride on their parent atoms, with aromatic  C—H = 0.93 Å, O—H = 0.85 Å and refined as riding on their parent atoms. The*U*_iso_(H) values were set at 1.2*U*_eq_(carrier atom) for all H atoms.

### Crystal data

**Table:**

[Ba_2_(C_5_H_3_N_2_O_2_)_2_(H_2_O)_7_](C_5_H_3_N_2_O_2_)_2_	*F*(000) = 868
*M**_r_* = 893.17	*D*_x_ = 2.009 Mg m^−^^3^
Monoclinic, *P*2_1_	Mo *K*α radiation, λ = 0.71073 Å
Hall symbol: P 2yb	Cell parameters from 4314 reflections
*a* = 7.5652 (10) Å	θ = 2.8–27.6°
*b* = 29.263 (3) Å	µ = 2.74 mm^−^^1^
*c* = 7.6067 (10) Å	*T* = 298 K
β = 118.741 (2)°	Block, colourless
*V* = 1476.5 (3) Å^3^	0.49 × 0.34 × 0.16 mm
*Z* = 2	

### Data collection

**Table:**

Bruker SMART APEX CCD area-detector diffractometer	4734 independent reflections
Radiation source: fine-focus sealed tube	4502 reflections with *I* > 2σ(*I*)
graphite	*R*_int_ = 0.036
Detector resolution: 0 pixels mm^-1^	θ_max_ = 25.0°, θ_min_ = 1.4°
φ and ω scans	*h* = −8→6
Absorption correction: multi-scan (*SADABS*; Bruker, 2002)	*k* = −34→32
*T*_min_ = 0.348, *T*_max_ = 0.669	*l* = −8→9
7342 measured reflections	

### Refinement

**Table:**

Refinement on *F*^2^	Hydrogen site location: inferred from neighbouring sites
Least-squares matrix: full	H-atom parameters constrained
*R*[*F*^2^ > 2σ(*F*^2^)] = 0.046	*w* = 1/[σ^2^(*F*_o_^2^) + (0.062*P*)^2^ + 10.4333*P*] where *P* = (*F*_o_^2^ + 2*F*_c_^2^)/3
*wR*(*F*^2^) = 0.121	(Δ/σ)_max_ < 0.001
*S* = 1.02	Δρ_max_ = 1.84 e Å^−^^3^
4734 reflections	Δρ_min_ = −2.62 e Å^−^^3^
406 parameters	Extinction correction: *SHELXL97* (Sheldrick, 2008)
1 restraint	Extinction coefficient: 0.00273 (13)
0 constraints	Absolute structure: Flack (1983), 2096 Friedel pairs
Primary atom site location: structure-invariant direct methods	Flack parameter: 0.04 (4)
Secondary atom site location: difference Fourier map	

### Fractional atomic coordinates and isotropic or equivalent isotropic displacement parameters (Å^2^)

**Table:**

	*x*	*y*	*z*	*U*_iso_*/*U*_eq_	
Ba1	0.77811 (9)	0.366622 (19)	0.36016 (9)	0.02014 (17)	
Ba2	1.10429 (9)	0.394536 (18)	0.03210 (9)	0.01982 (16)	
N1	0.8301 (16)	0.2639 (3)	0.3451 (15)	0.026 (2)	
N2	0.888 (2)	0.1707 (4)	0.308 (2)	0.043 (3)	
N3	1.0849 (15)	0.4975 (4)	0.0828 (17)	0.028 (2)	
N4	1.045 (2)	0.5910 (4)	0.137 (2)	0.045 (3)	
N5	0.6725 (18)	0.5151 (4)	0.6133 (18)	0.033 (3)	
N6	0.5658 (18)	0.6073 (4)	0.610 (2)	0.039 (3)	
N7	0.3586 (18)	0.2445 (4)	0.9189 (19)	0.032 (3)	
N8	0.400 (2)	0.1538 (4)	0.828 (2)	0.052 (4)	
O1	0.8792 (14)	0.3243 (3)	0.0951 (15)	0.034 (2)	
O2	0.9048 (17)	0.2671 (3)	−0.0797 (14)	0.039 (2)	
O3	0.8422 (13)	0.4370 (3)	0.1352 (14)	0.033 (2)	
O4	0.6620 (16)	0.4940 (3)	0.1590 (18)	0.045 (3)	
O5	0.4326 (14)	0.4487 (3)	0.6353 (16)	0.040 (2)	
O6	0.2265 (14)	0.5026 (4)	0.6485 (15)	0.043 (3)	
O7	0.3818 (16)	0.3133 (3)	0.6871 (15)	0.042 (2)	
O8	0.4035 (18)	0.2619 (4)	0.4835 (15)	0.051 (3)	
O9	1.1777 (11)	0.3806 (3)	0.4331 (11)	0.0267 (17)	
H9A	1.2515	0.3573	0.4865	0.032*	
H9B	1.2312	0.4040	0.5061	0.032*	
O10	0.6963 (12)	0.3775 (3)	0.6959 (12)	0.0278 (18)	
H10A	0.6450	0.3534	0.7149	0.033*	
H10B	0.6182	0.4000	0.6777	0.033*	
O11	0.4867 (12)	0.4342 (3)	0.2934 (13)	0.0288 (19)	
H11A	0.5066	0.4577	0.2396	0.035*	
H11B	0.4921	0.4421	0.4035	0.035*	
O12	0.4417 (12)	0.3857 (3)	−0.0462 (12)	0.031 (2)	
H12A	0.4241	0.3637	−0.1259	0.038*	
H12B	0.4614	0.4102	−0.0945	0.038*	
O13	0.9582 (12)	0.4406 (3)	0.6526 (12)	0.0294 (18)	
H13A	0.8706	0.4607	0.6372	0.035*	
H13B	1.0541	0.4533	0.6426	0.035*	
O14	0.3952 (12)	0.3203 (3)	0.2065 (13)	0.0296 (19)	
H14A	0.3838	0.3071	0.3001	0.036*	
H14B	0.3806	0.3006	0.1182	0.036*	
O15	1.0377 (12)	0.3263 (3)	0.7417 (12)	0.0271 (18)	
H15A	1.1486	0.3182	0.7481	0.032*	
H15B	0.9823	0.3029	0.7608	0.032*	
C1	0.8864 (17)	0.2828 (4)	0.0620 (17)	0.021 (2)	
C2	0.874 (2)	0.2485 (4)	0.2049 (19)	0.027 (3)	
C3	0.896 (2)	0.2017 (4)	0.184 (2)	0.036 (3)	
H3	0.9171	0.1918	0.0790	0.043*	
C5	0.847 (3)	0.1868 (6)	0.449 (3)	0.046 (4)	
H5	0.8360	0.1666	0.5373	0.056*	
C6	0.822 (2)	0.2336 (5)	0.467 (2)	0.036 (3)	
H6	0.7979	0.2435	0.5695	0.043*	
C7	0.804 (2)	0.4782 (5)	0.1384 (19)	0.030 (3)	
C8	0.944 (2)	0.5124 (4)	0.1270 (19)	0.030 (3)	
C9	0.923 (2)	0.5590 (5)	0.149 (2)	0.039 (3)	
H9	0.8193	0.5685	0.1736	0.047*	
C11	1.187 (2)	0.5754 (5)	0.100 (3)	0.041 (4)	
H11	1.2768	0.5960	0.0932	0.050*	
C12	1.207 (2)	0.5296 (5)	0.071 (2)	0.034 (3)	
H12	1.3088	0.5205	0.0432	0.041*	
C13	0.3801 (19)	0.4890 (4)	0.640 (2)	0.026 (3)	
C14	0.5088 (18)	0.5268 (4)	0.6264 (17)	0.022 (2)	
C15	0.461 (2)	0.5730 (5)	0.624 (2)	0.036 (3)	
H15	0.3458	0.5801	0.6341	0.043*	
C17	0.727 (2)	0.5944 (5)	0.592 (2)	0.036 (3)	
H17	0.8058	0.6168	0.5771	0.043*	
C18	0.779 (2)	0.5490 (5)	0.595 (2)	0.041 (4)	
H18	0.8932	0.5419	0.5840	0.049*	
C19	0.396 (2)	0.2731 (4)	0.636 (2)	0.028 (3)	
C20	0.3868 (17)	0.2347 (4)	0.7658 (19)	0.026 (3)	
C21	0.409 (2)	0.1888 (5)	0.723 (2)	0.038 (3)	
H21	0.4321	0.1828	0.6159	0.045*	
C23	0.363 (2)	0.1655 (6)	0.977 (2)	0.045 (4)	
H23	0.3494	0.1424	1.0533	0.054*	
C24	0.346 (2)	0.2099 (5)	1.022 (2)	0.043 (4)	
H24	0.3232	0.2159	1.1296	0.051*	

### Atomic displacement parameters (Å^2^)

**Table:**

	*U*^11^	*U*^22^	*U*^33^	*U*^12^	*U*^13^	*U*^23^
Ba1	0.0175 (3)	0.0207 (3)	0.0234 (3)	0.0000 (3)	0.0108 (3)	−0.0010 (3)
Ba2	0.0184 (3)	0.0207 (3)	0.0216 (3)	0.0006 (3)	0.0106 (3)	−0.0005 (3)
N1	0.036 (6)	0.013 (5)	0.030 (6)	−0.005 (4)	0.017 (5)	−0.003 (4)
N2	0.053 (8)	0.023 (6)	0.065 (8)	0.011 (5)	0.038 (7)	0.007 (6)
N3	0.029 (6)	0.029 (6)	0.041 (6)	0.006 (5)	0.029 (5)	0.011 (5)
N4	0.053 (8)	0.019 (6)	0.073 (9)	−0.005 (5)	0.039 (7)	0.001 (6)
N5	0.028 (6)	0.032 (6)	0.042 (7)	0.000 (5)	0.018 (5)	0.005 (5)
N6	0.042 (7)	0.015 (6)	0.065 (9)	−0.006 (5)	0.028 (6)	0.002 (5)
N7	0.036 (6)	0.029 (6)	0.038 (6)	0.005 (5)	0.025 (6)	0.006 (5)
N8	0.096 (11)	0.018 (6)	0.049 (8)	0.005 (6)	0.041 (8)	0.007 (6)
O1	0.042 (5)	0.019 (5)	0.053 (6)	−0.002 (4)	0.031 (5)	−0.001 (4)
O2	0.076 (7)	0.021 (5)	0.036 (5)	−0.003 (5)	0.042 (6)	0.001 (4)
O3	0.040 (5)	0.022 (5)	0.055 (6)	0.006 (4)	0.037 (5)	0.010 (4)
O4	0.047 (6)	0.028 (5)	0.082 (8)	0.007 (4)	0.049 (6)	0.007 (5)
O5	0.043 (6)	0.030 (5)	0.060 (6)	−0.002 (4)	0.034 (5)	−0.008 (5)
O6	0.043 (6)	0.035 (6)	0.070 (8)	−0.003 (4)	0.042 (6)	0.001 (5)
O7	0.052 (6)	0.037 (5)	0.046 (6)	0.013 (5)	0.031 (5)	−0.001 (5)
O8	0.092 (9)	0.038 (6)	0.042 (6)	0.002 (6)	0.047 (7)	0.006 (5)
O9	0.020 (4)	0.033 (4)	0.025 (4)	0.000 (3)	0.008 (3)	−0.004 (3)
O10	0.028 (4)	0.027 (5)	0.036 (4)	0.000 (3)	0.022 (4)	0.001 (4)
O11	0.023 (4)	0.027 (5)	0.039 (5)	−0.004 (3)	0.017 (4)	−0.008 (4)
O12	0.041 (5)	0.031 (5)	0.030 (4)	−0.006 (4)	0.024 (4)	0.002 (4)
O13	0.028 (4)	0.025 (4)	0.041 (5)	0.000 (3)	0.021 (4)	0.000 (4)
O14	0.037 (5)	0.016 (4)	0.041 (5)	0.004 (3)	0.023 (4)	0.002 (4)
O15	0.031 (5)	0.020 (4)	0.031 (5)	0.003 (3)	0.016 (4)	−0.002 (3)
C1	0.020 (6)	0.027 (7)	0.018 (6)	−0.009 (5)	0.011 (5)	−0.002 (5)
C2	0.041 (8)	0.021 (6)	0.028 (7)	−0.009 (5)	0.024 (6)	−0.008 (5)
C3	0.070 (10)	0.007 (6)	0.058 (9)	−0.001 (6)	0.052 (9)	0.002 (6)
C5	0.049 (9)	0.037 (9)	0.055 (10)	−0.001 (8)	0.027 (8)	0.014 (8)
C6	0.045 (8)	0.041 (8)	0.023 (7)	0.004 (7)	0.018 (6)	0.005 (6)
C7	0.033 (7)	0.030 (7)	0.032 (7)	0.004 (6)	0.020 (6)	0.006 (6)
C8	0.042 (8)	0.017 (6)	0.029 (7)	0.000 (5)	0.016 (6)	0.001 (5)
C9	0.049 (9)	0.033 (8)	0.049 (9)	−0.002 (6)	0.035 (8)	−0.003 (6)
C11	0.040 (8)	0.029 (8)	0.059 (10)	−0.006 (7)	0.027 (8)	0.013 (7)
C12	0.032 (7)	0.042 (8)	0.035 (8)	0.004 (6)	0.021 (6)	0.002 (6)
C13	0.024 (6)	0.029 (7)	0.030 (7)	−0.009 (5)	0.016 (6)	−0.008 (5)
C14	0.028 (6)	0.016 (6)	0.024 (6)	0.002 (5)	0.015 (5)	0.001 (5)
C15	0.044 (8)	0.027 (7)	0.048 (9)	0.004 (6)	0.033 (7)	0.001 (6)
C17	0.043 (8)	0.021 (7)	0.049 (9)	−0.008 (6)	0.027 (7)	0.006 (6)
C18	0.032 (7)	0.039 (8)	0.063 (10)	−0.011 (6)	0.031 (8)	−0.003 (7)
C19	0.037 (7)	0.029 (7)	0.025 (7)	0.007 (5)	0.020 (6)	0.003 (5)
C20	0.018 (6)	0.025 (7)	0.035 (7)	0.004 (5)	0.011 (5)	0.000 (5)
C21	0.053 (9)	0.025 (7)	0.039 (8)	0.003 (6)	0.025 (7)	0.005 (6)
C23	0.056 (10)	0.042 (9)	0.034 (8)	−0.003 (7)	0.019 (8)	0.010 (7)
C24	0.043 (9)	0.054 (10)	0.037 (8)	0.000 (7)	0.024 (7)	−0.006 (7)

### Geometric parameters (Å, °)

**Table:**

Ba1—O1	2.769 (9)	O9—H9A	0.8500
Ba1—O11	2.821 (8)	O9—H9B	0.8500
Ba1—O9	2.829 (7)	O10—Ba2^iii^	2.954 (8)
Ba1—O15	2.862 (8)	O10—H10A	0.8500
Ba1—O3	2.865 (8)	O10—H10B	0.8501
Ba1—O14	2.888 (8)	O11—Ba2^iv^	2.853 (8)
Ba1—O10	2.920 (7)	O11—H11A	0.8499
Ba1—O13	2.930 (8)	O11—H11B	0.8500
Ba1—O12	2.963 (8)	O12—Ba2^iv^	2.898 (8)
Ba1—N1	3.041 (10)	O12—H12A	0.8499
Ba2—O3	2.754 (8)	O12—H12B	0.8501
Ba2—O15^i^	2.838 (8)	O13—Ba2^iii^	2.882 (8)
Ba2—O11^ii^	2.853 (8)	O13—H13A	0.8499
Ba2—O1	2.853 (8)	O13—H13B	0.8499
Ba2—O9	2.854 (7)	O14—Ba2^iv^	2.919 (8)
Ba2—O13^i^	2.882 (8)	O14—H14A	0.8499
Ba2—O12^ii^	2.898 (8)	O14—H14B	0.8501
Ba2—O14^ii^	2.919 (8)	O15—Ba2^iii^	2.838 (8)
Ba2—O10^i^	2.954 (8)	O15—H15A	0.8500
Ba2—N3	3.050 (11)	O15—H15B	0.8501
N1—C6	1.305 (17)	C1—C2	1.515 (16)
N1—C2	1.336 (16)	C2—C3	1.401 (17)
N2—C3	1.332 (17)	C3—H3	0.9300
N2—C5	1.34 (2)	C5—C6	1.40 (2)
N3—C8	1.334 (17)	C5—H5	0.9300
N3—C12	1.351 (17)	C6—H6	0.9300
N4—C11	1.32 (2)	C7—C8	1.489 (18)
N4—C9	1.349 (18)	C8—C9	1.394 (19)
N5—C18	1.326 (18)	C9—H9	0.9300
N5—C14	1.334 (16)	C11—C12	1.38 (2)
N6—C15	1.317 (17)	C11—H11	0.9300
N6—C17	1.341 (18)	C12—H12	0.9300
N7—C24	1.312 (19)	C13—C14	1.509 (16)
N7—C20	1.313 (16)	C14—C15	1.400 (18)
N8—C21	1.320 (18)	C15—H15	0.9300
N8—C23	1.331 (19)	C17—C18	1.39 (2)
O1—C1	1.246 (14)	C17—H17	0.9300
O2—C1	1.240 (14)	C18—H18	0.9300
O3—C7	1.243 (15)	C19—C20	1.519 (18)
O4—C7	1.248 (16)	C20—C21	1.413 (19)
O5—C13	1.249 (15)	C21—H21	0.9300
O6—C13	1.259 (15)	C23—C24	1.37 (2)
O7—C19	1.260 (16)	C23—H23	0.9300
O8—C19	1.233 (15)	C24—H24	0.9300
			
O1—Ba1—O11	130.4 (3)	O13^i^—Ba2—Ba1^ii^	115.01 (16)
O1—Ba1—O9	64.2 (2)	O12^ii^—Ba2—Ba1^ii^	39.69 (16)
O11—Ba1—O9	127.1 (2)	O14^ii^—Ba2—Ba1^ii^	38.27 (16)
O1—Ba1—O15	105.0 (2)	O10^i^—Ba2—Ba1^ii^	150.97 (15)
O11—Ba1—O15	124.6 (2)	N3—Ba2—Ba1^ii^	102.6 (2)
O9—Ba1—O15	73.3 (2)	Ba1—Ba2—Ba1^ii^	116.58 (2)
O1—Ba1—O3	72.6 (2)	C6—N1—C2	116.8 (11)
O11—Ba1—O3	74.4 (2)	C6—N1—Ba1	126.5 (9)
O9—Ba1—O3	62.7 (2)	C2—N1—Ba1	116.6 (8)
O15—Ba1—O3	132.2 (3)	C3—N2—C5	115.8 (12)
O1—Ba1—O14	94.0 (2)	C8—N3—C12	116.4 (12)
O11—Ba1—O14	73.3 (2)	C8—N3—Ba2	116.6 (8)
O9—Ba1—O14	156.4 (2)	C12—N3—Ba2	127.0 (8)
O15—Ba1—O14	106.4 (2)	C11—N4—C9	115.5 (12)
O3—Ba1—O14	121.4 (3)	C18—N5—C14	116.8 (13)
O1—Ba1—O10	159.1 (2)	C15—N6—C17	114.2 (11)
O11—Ba1—O10	66.7 (2)	C24—N7—C20	116.9 (13)
O9—Ba1—O10	118.0 (2)	C21—N8—C23	114.0 (13)
O15—Ba1—O10	59.2 (2)	C1—O1—Ba1	129.6 (7)
O3—Ba1—O10	127.8 (2)	C1—O1—Ba2	125.5 (7)
O14—Ba1—O10	79.2 (2)	Ba1—O1—Ba2	101.3 (3)
O1—Ba1—O13	133.9 (2)	C7—O3—Ba2	130.5 (8)
O11—Ba1—O13	69.9 (2)	C7—O3—Ba1	125.3 (8)
O9—Ba1—O13	71.4 (2)	Ba2—O3—Ba1	101.4 (3)
O15—Ba1—O13	72.1 (2)	Ba1—O9—Ba2	99.8 (2)
O3—Ba1—O13	76.8 (2)	Ba1—O9—H9A	111.8
O14—Ba1—O13	131.6 (2)	Ba2—O9—H9A	111.8
O10—Ba1—O13	58.1 (2)	Ba1—O9—H9B	111.8
O1—Ba1—O12	74.0 (3)	Ba2—O9—H9B	111.8
O11—Ba1—O12	58.3 (2)	H9A—O9—H9B	109.6
O9—Ba1—O12	118.6 (2)	Ba1—O10—Ba2^iii^	101.7 (2)
O15—Ba1—O12	164.2 (2)	Ba1—O10—H10A	111.4
O3—Ba1—O12	63.1 (3)	Ba2^iii^—O10—H10A	111.4
O14—Ba1—O12	58.4 (2)	Ba1—O10—H10B	111.4
O10—Ba1—O12	117.1 (2)	Ba2^iii^—O10—H10B	111.4
O13—Ba1—O12	120.4 (2)	H10A—O10—H10B	109.3
O1—Ba1—N1	56.2 (3)	Ba1—O11—Ba2^iv^	106.4 (3)
O11—Ba1—N1	142.2 (3)	Ba1—O11—H11A	110.4
O9—Ba1—N1	90.3 (3)	Ba2^iv^—O11—H11A	110.4
O15—Ba1—N1	65.9 (2)	Ba1—O11—H11B	110.4
O3—Ba1—N1	128.8 (3)	Ba2^iv^—O11—H11B	110.4
O14—Ba1—N1	69.1 (3)	H11A—O11—H11B	108.7
O10—Ba1—N1	103.1 (3)	Ba2^iv^—O12—Ba1	101.6 (2)
O13—Ba1—N1	137.6 (3)	Ba2^iv^—O12—H12A	111.2
O12—Ba1—N1	102.1 (3)	Ba1—O12—H12A	111.2
O1—Ba1—Ba2	40.06 (17)	Ba2^iv^—O12—H12B	111.6
O11—Ba1—Ba2	112.03 (18)	Ba1—O12—H12B	111.6
O9—Ba1—Ba2	40.31 (15)	H12A—O12—H12B	109.3
O15—Ba1—Ba2	110.94 (17)	Ba2^iii^—O13—Ba1	103.2 (2)
O3—Ba1—Ba2	38.39 (16)	Ba2^iii^—O13—H13A	111.0
O14—Ba1—Ba2	126.27 (17)	Ba1—O13—H13A	111.0
O10—Ba1—Ba2	154.02 (16)	Ba2^iii^—O13—H13B	111.2
O13—Ba1—Ba2	96.42 (16)	Ba1—O13—H13B	111.1
O12—Ba1—Ba2	78.79 (15)	H13A—O13—H13B	109.2
N1—Ba1—Ba2	92.6 (2)	Ba1—O14—Ba2^iv^	103.0 (2)
O1—Ba1—Ba2^iv^	108.1 (2)	Ba1—O14—H14A	111.1
O11—Ba1—Ba2^iv^	37.04 (16)	Ba2^iv^—O14—H14A	111.1
O9—Ba1—Ba2^iv^	153.20 (16)	Ba1—O14—H14B	111.2
O15—Ba1—Ba2^iv^	132.27 (17)	Ba2^iv^—O14—H14B	111.2
O3—Ba1—Ba2^iv^	90.58 (18)	H14A—O14—H14B	109.2
O14—Ba1—Ba2^iv^	38.76 (15)	Ba2^iii^—O15—Ba1	106.1 (2)
O10—Ba1—Ba2^iv^	78.88 (15)	Ba2^iii^—O15—H15A	110.5
O13—Ba1—Ba2^iv^	105.87 (16)	Ba1—O15—H15A	110.5
O12—Ba1—Ba2^iv^	38.66 (15)	Ba2^iii^—O15—H15B	110.5
N1—Ba1—Ba2^iv^	106.8 (2)	Ba1—O15—H15B	110.5
Ba2—Ba1—Ba2^iv^	116.58 (2)	H15A—O15—H15B	108.7
O3—Ba2—O15^i^	131.0 (3)	O2—C1—O1	124.8 (11)
O3—Ba2—O11^ii^	104.2 (2)	O2—C1—C2	116.8 (10)
O15^i^—Ba2—O11^ii^	124.7 (2)	O1—C1—C2	118.4 (10)
O3—Ba2—O1	73.0 (2)	N1—C2—C3	120.7 (11)
O15^i^—Ba2—O1	74.1 (2)	N1—C2—C1	118.4 (11)
O11^ii^—Ba2—O1	131.6 (3)	C3—C2—C1	120.8 (10)
O3—Ba2—O9	63.7 (2)	N2—C3—C2	122.4 (12)
O15^i^—Ba2—O9	127.0 (2)	N2—C3—H3	118.8
O11^ii^—Ba2—O9	72.7 (2)	C2—C3—H3	118.8
O1—Ba2—O9	62.8 (3)	N2—C5—C6	121.3 (14)
O3—Ba2—O13^i^	95.3 (2)	N2—C5—H5	119.3
O15^i^—Ba2—O13^i^	73.2 (2)	C6—C5—H5	119.3
O11^ii^—Ba2—O13^i^	106.2 (2)	N1—C6—C5	122.8 (13)
O1—Ba2—O13^i^	122.2 (3)	N1—C6—H6	118.6
O9—Ba2—O13^i^	157.1 (2)	C5—C6—H6	118.6
O3—Ba2—O12^ii^	157.6 (2)	O3—C7—O4	125.7 (12)
O15^i^—Ba2—O12^ii^	67.8 (2)	O3—C7—C8	118.2 (11)
O11^ii^—Ba2—O12^ii^	58.8 (2)	O4—C7—C8	116.1 (12)
O1—Ba2—O12^ii^	128.8 (2)	N3—C8—C9	120.3 (12)
O9—Ba2—O12^ii^	118.0 (2)	N3—C8—C7	118.1 (11)
O13^i^—Ba2—O12^ii^	77.7 (2)	C9—C8—C7	121.5 (12)
O3—Ba2—O14^ii^	134.1 (2)	N4—C9—C8	123.2 (13)
O15^i^—Ba2—O14^ii^	69.0 (2)	N4—C9—H9	118.4
O11^ii^—Ba2—O14^ii^	72.3 (2)	C8—C9—H9	118.4
O1—Ba2—O14^ii^	76.6 (2)	N4—C11—C12	122.5 (13)
O9—Ba2—O14^ii^	72.0 (2)	N4—C11—H11	118.8
O13^i^—Ba2—O14^ii^	130.2 (2)	C12—C11—H11	118.8
O12^ii^—Ba2—O14^ii^	58.7 (2)	N3—C12—C11	122.2 (12)
O3—Ba2—O10^i^	74.2 (2)	N3—C12—H12	118.9
O15^i^—Ba2—O10^i^	59.1 (2)	C11—C12—H12	118.9
O11^ii^—Ba2—O10^i^	163.6 (2)	O5—C13—O6	127.8 (12)
O1—Ba2—O10^i^	64.2 (2)	O5—C13—C14	117.7 (11)
O9—Ba2—O10^i^	119.1 (2)	O6—C13—C14	114.4 (11)
O13^i^—Ba2—O10^i^	58.3 (2)	N5—C14—C15	119.5 (12)
O12^ii^—Ba2—O10^i^	117.6 (2)	N5—C14—C13	118.1 (11)
O14^ii^—Ba2—O10^i^	120.9 (2)	C15—C14—C13	122.5 (11)
O3—Ba2—N3	55.5 (2)	N6—C15—C14	124.9 (12)
O15^i^—Ba2—N3	142.4 (3)	N6—C15—H15	117.6
O11^ii^—Ba2—N3	66.7 (3)	C14—C15—H15	117.6
O1—Ba2—N3	128.4 (2)	N6—C17—C18	122.2 (12)
O9—Ba2—N3	90.1 (3)	N6—C17—H17	118.9
O13^i^—Ba2—N3	69.2 (3)	C18—C17—H17	118.9
O12^ii^—Ba2—N3	102.4 (3)	N5—C18—C17	122.4 (13)
O14^ii^—Ba2—N3	138.6 (3)	N5—C18—H18	118.8
O10^i^—Ba2—N3	100.5 (3)	C17—C18—H18	118.8
O3—Ba2—Ba1	40.23 (16)	O8—C19—O7	126.1 (12)
O15^i^—Ba2—Ba1	112.16 (17)	O8—C19—C20	116.8 (11)
O11^ii^—Ba2—Ba1	109.98 (17)	O7—C19—C20	116.9 (11)
O1—Ba2—Ba1	38.65 (17)	N7—C20—C21	119.9 (12)
O9—Ba2—Ba1	39.87 (15)	N7—C20—C19	119.6 (11)
O13^i^—Ba2—Ba1	127.78 (16)	C21—C20—C19	120.5 (11)
O12^ii^—Ba2—Ba1	154.19 (16)	N8—C21—C20	123.6 (13)
O14^ii^—Ba2—Ba1	96.38 (17)	N8—C21—H21	118.2
O10^i^—Ba2—Ba1	79.64 (15)	C20—C21—H21	118.2
N3—Ba2—Ba1	92.17 (18)	N8—C23—C24	122.9 (14)
O3—Ba2—Ba1^ii^	134.1 (2)	N8—C23—H23	118.6
O15^i^—Ba2—Ba1^ii^	91.90 (17)	C24—C23—H23	118.6
O11^ii^—Ba2—Ba1^ii^	36.55 (16)	N7—C24—C23	122.6 (14)
O1—Ba2—Ba1^ii^	112.37 (18)	N7—C24—H24	118.7
O9—Ba2—Ba1^ii^	78.29 (15)	C23—C24—H24	118.7
			
O1—Ba1—Ba2—O3	139.0 (4)	O11^ii^—Ba2—O1—Ba1	67.8 (4)
O11—Ba1—Ba2—O3	11.7 (4)	O9—Ba2—O1—Ba1	42.4 (3)
O9—Ba1—Ba2—O3	−110.2 (4)	O13^i^—Ba2—O1—Ba1	−111.8 (3)
O15—Ba1—Ba2—O3	−132.5 (4)	O12^ii^—Ba2—O1—Ba1	147.6 (2)
O14—Ba1—Ba2—O3	96.6 (4)	O14^ii^—Ba2—O1—Ba1	118.8 (3)
O10—Ba1—Ba2—O3	−70.1 (5)	O10^i^—Ba2—O1—Ba1	−106.6 (3)
O13—Ba1—Ba2—O3	−59.2 (3)	N3—Ba2—O1—Ba1	−23.8 (5)
O12—Ba1—Ba2—O3	60.6 (4)	Ba1^ii^—Ba2—O1—Ba1	105.1 (2)
N1—Ba1—Ba2—O3	162.4 (4)	O15^i^—Ba2—O3—C7	−123.5 (11)
Ba2^iv^—Ba1—Ba2—O3	52.2 (3)	O11^ii^—Ba2—O3—C7	57.0 (12)
O1—Ba1—Ba2—O15^i^	10.9 (3)	O1—Ba2—O3—C7	−173.3 (12)
O11—Ba1—Ba2—O15^i^	−116.4 (2)	O9—Ba2—O3—C7	119.2 (12)
O9—Ba1—Ba2—O15^i^	121.7 (3)	O13^i^—Ba2—O3—C7	−51.2 (11)
O15—Ba1—Ba2—O15^i^	99.5 (3)	O12^ii^—Ba2—O3—C7	19.2 (15)
O3—Ba1—Ba2—O15^i^	−128.1 (4)	O14^ii^—Ba2—O3—C7	136.0 (11)
O14—Ba1—Ba2—O15^i^	−31.5 (3)	O10^i^—Ba2—O3—C7	−106.1 (11)
O10—Ba1—Ba2—O15^i^	161.8 (4)	N3—Ba2—O3—C7	9.1 (11)
O13—Ba1—Ba2—O15^i^	172.8 (2)	Ba1—Ba2—O3—C7	161.3 (13)
O12—Ba1—Ba2—O15^i^	−67.5 (2)	Ba1^ii^—Ba2—O3—C7	81.7 (12)
N1—Ba1—Ba2—O15^i^	34.3 (3)	O15^i^—Ba2—O3—Ba1	75.2 (4)
Ba2^iv^—Ba1—Ba2—O15^i^	−75.88 (18)	O11^ii^—Ba2—O3—Ba1	−104.3 (3)
O1—Ba1—Ba2—O11^ii^	−132.5 (4)	O1—Ba2—O3—Ba1	25.4 (3)
O11—Ba1—Ba2—O11^ii^	100.2 (3)	O9—Ba2—O3—Ba1	−42.1 (3)
O9—Ba1—Ba2—O11^ii^	−21.7 (3)	O13^i^—Ba2—O3—Ba1	147.5 (3)
O15—Ba1—Ba2—O11^ii^	−43.9 (2)	O12^ii^—Ba2—O3—Ba1	−142.2 (5)
O3—Ba1—Ba2—O11^ii^	88.5 (4)	O14^ii^—Ba2—O3—Ba1	−25.3 (5)
O14—Ba1—Ba2—O11^ii^	−174.9 (3)	O10^i^—Ba2—O3—Ba1	92.6 (3)
O10—Ba1—Ba2—O11^ii^	18.4 (4)	N3—Ba2—O3—Ba1	−152.2 (4)
O13—Ba1—Ba2—O11^ii^	29.4 (2)	Ba1^ii^—Ba2—O3—Ba1	−79.6 (3)
O12—Ba1—Ba2—O11^ii^	149.1 (2)	O1—Ba1—O3—C7	171.1 (11)
N1—Ba1—Ba2—O11^ii^	−109.1 (3)	O11—Ba1—O3—C7	28.6 (10)
Ba2^iv^—Ba1—Ba2—O11^ii^	140.71 (17)	O9—Ba1—O3—C7	−119.5 (11)
O11—Ba1—Ba2—O1	−127.3 (4)	O15—Ba1—O3—C7	−94.2 (11)
O9—Ba1—Ba2—O1	110.8 (4)	O14—Ba1—O3—C7	87.2 (11)
O15—Ba1—Ba2—O1	88.6 (3)	O10—Ba1—O3—C7	−14.0 (11)
O3—Ba1—Ba2—O1	−139.0 (4)	O13—Ba1—O3—C7	−43.8 (10)
O14—Ba1—Ba2—O1	−42.4 (4)	O12—Ba1—O3—C7	90.6 (10)
O10—Ba1—Ba2—O1	150.9 (5)	N1—Ba1—O3—C7	174.5 (10)
O13—Ba1—Ba2—O1	161.9 (3)	Ba2—Ba1—O3—C7	−162.6 (12)
O12—Ba1—Ba2—O1	−78.4 (4)	Ba2^iv^—Ba1—O3—C7	62.3 (10)
N1—Ba1—Ba2—O1	23.4 (4)	O1—Ba1—O3—Ba2	−26.3 (3)
Ba2^iv^—Ba1—Ba2—O1	−86.8 (3)	O11—Ba1—O3—Ba2	−168.8 (3)
O1—Ba1—Ba2—O9	−110.8 (4)	O9—Ba1—O3—Ba2	43.1 (3)
O11—Ba1—Ba2—O9	121.9 (3)	O15—Ba1—O3—Ba2	68.4 (4)
O15—Ba1—Ba2—O9	−22.3 (3)	O14—Ba1—O3—Ba2	−110.2 (3)
O3—Ba1—Ba2—O9	110.2 (4)	O10—Ba1—O3—Ba2	148.6 (2)
O14—Ba1—Ba2—O9	−153.2 (3)	O13—Ba1—O3—Ba2	118.8 (3)
O10—Ba1—Ba2—O9	40.1 (4)	O12—Ba1—O3—Ba2	−106.7 (3)
O13—Ba1—Ba2—O9	51.0 (3)	N1—Ba1—O3—Ba2	−22.9 (5)
O12—Ba1—Ba2—O9	170.8 (3)	Ba2^iv^—Ba1—O3—Ba2	−135.1 (2)
N1—Ba1—Ba2—O9	−87.4 (3)	O1—Ba1—O9—Ba2	41.9 (2)
Ba2^iv^—Ba1—Ba2—O9	162.4 (3)	O11—Ba1—O9—Ba2	−80.6 (3)
O1—Ba1—Ba2—O13^i^	96.4 (4)	O15—Ba1—O9—Ba2	158.3 (3)
O11—Ba1—Ba2—O13^i^	−30.9 (3)	O3—Ba1—O9—Ba2	−41.0 (2)
O9—Ba1—Ba2—O13^i^	−152.8 (3)	O14—Ba1—O9—Ba2	65.4 (6)
O15—Ba1—Ba2—O13^i^	−175.1 (3)	O10—Ba1—O9—Ba2	−161.4 (2)
O3—Ba1—Ba2—O13^i^	−42.6 (4)	O13—Ba1—O9—Ba2	−125.4 (3)
O14—Ba1—Ba2—O13^i^	54.0 (3)	O12—Ba1—O9—Ba2	−10.3 (4)
O10—Ba1—Ba2—O13^i^	−112.8 (4)	N1—Ba1—O9—Ba2	93.6 (3)
O13—Ba1—Ba2—O13^i^	−101.8 (3)	Ba2^iv^—Ba1—O9—Ba2	−36.9 (5)
O12—Ba1—Ba2—O13^i^	18.0 (3)	O3—Ba2—O9—Ba1	42.5 (2)
N1—Ba1—Ba2—O13^i^	119.8 (3)	O15^i^—Ba2—O9—Ba1	−80.5 (3)
Ba2^iv^—Ba1—Ba2—O13^i^	9.6 (2)	O11^ii^—Ba2—O9—Ba1	158.7 (3)
O1—Ba1—Ba2—O12^ii^	−73.5 (5)	O1—Ba2—O9—Ba1	−41.0 (2)
O11—Ba1—Ba2—O12^ii^	159.2 (4)	O13^i^—Ba2—O9—Ba1	67.9 (6)
O9—Ba1—Ba2—O12^ii^	37.3 (5)	O12^ii^—Ba2—O9—Ba1	−162.6 (2)
O15—Ba1—Ba2—O12^ii^	15.0 (4)	O14^ii^—Ba2—O9—Ba1	−124.8 (3)
O3—Ba1—Ba2—O12^ii^	147.5 (5)	O10^i^—Ba2—O9—Ba1	−8.9 (3)
O14—Ba1—Ba2—O12^ii^	−115.9 (4)	N3—Ba2—O9—Ba1	93.2 (3)
O10—Ba1—Ba2—O12^ii^	77.3 (5)	Ba1^ii^—Ba2—O9—Ba1	−163.9 (2)
O13—Ba1—Ba2—O12^ii^	88.3 (4)	O1—Ba1—O10—Ba2^iii^	86.2 (7)
O12—Ba1—Ba2—O12^ii^	−151.9 (6)	O11—Ba1—O10—Ba2^iii^	−125.2 (3)
N1—Ba1—Ba2—O12^ii^	−50.2 (4)	O9—Ba1—O10—Ba2^iii^	−4.2 (3)
Ba2^iv^—Ba1—Ba2—O12^ii^	−160.3 (4)	O15—Ba1—O10—Ba2^iii^	41.9 (2)
O1—Ba1—Ba2—O14^ii^	−59.0 (3)	O3—Ba1—O10—Ba2^iii^	−79.9 (3)
O11—Ba1—Ba2—O14^ii^	173.7 (2)	O14—Ba1—O10—Ba2^iii^	158.5 (3)
O9—Ba1—Ba2—O14^ii^	51.8 (3)	O13—Ba1—O10—Ba2^iii^	−45.2 (2)
O15—Ba1—Ba2—O14^ii^	29.5 (2)	O12—Ba1—O10—Ba2^iii^	−155.7 (2)
O3—Ba1—Ba2—O14^ii^	162.0 (4)	N1—Ba1—O10—Ba2^iii^	93.2 (3)
O14—Ba1—Ba2—O14^ii^	−101.4 (3)	Ba2—Ba1—O10—Ba2^iii^	−32.3 (5)
O10—Ba1—Ba2—O14^ii^	91.9 (4)	Ba2^iv^—Ba1—O10—Ba2^iii^	−161.9 (2)
O13—Ba1—Ba2—O14^ii^	102.8 (2)	O1—Ba1—O11—Ba2^iv^	63.1 (4)
O12—Ba1—Ba2—O14^ii^	−137.4 (2)	O9—Ba1—O11—Ba2^iv^	148.9 (2)
N1—Ba1—Ba2—O14^ii^	−35.6 (3)	O15—Ba1—O11—Ba2^iv^	−116.3 (3)
Ba2^iv^—Ba1—Ba2—O14^ii^	−145.83 (17)	O3—Ba1—O11—Ba2^iv^	112.9 (3)
O1—Ba1—Ba2—O10^i^	61.3 (3)	O14—Ba1—O11—Ba2^iv^	−17.6 (2)
O11—Ba1—Ba2—O10^i^	−66.0 (2)	O10—Ba1—O11—Ba2^iv^	−102.8 (3)
O9—Ba1—Ba2—O10^i^	172.1 (3)	O13—Ba1—O11—Ba2^iv^	−165.8 (3)
O15—Ba1—Ba2—O10^i^	149.8 (2)	O12—Ba1—O11—Ba2^iv^	45.1 (3)
O3—Ba1—Ba2—O10^i^	−77.7 (3)	N1—Ba1—O11—Ba2^iv^	−21.6 (6)
O14—Ba1—Ba2—O10^i^	18.9 (2)	Ba2—Ba1—O11—Ba2^iv^	105.4 (2)
O10—Ba1—Ba2—O10^i^	−147.8 (5)	O1—Ba1—O12—Ba2^iv^	151.1 (3)
O13—Ba1—Ba2—O10^i^	−136.9 (2)	O11—Ba1—O12—Ba2^iv^	−43.1 (2)
O12—Ba1—Ba2—O10^i^	−17.1 (2)	O9—Ba1—O12—Ba2^iv^	−161.2 (2)
N1—Ba1—Ba2—O10^i^	84.7 (3)	O15—Ba1—O12—Ba2^iv^	62.6 (9)
Ba2^iv^—Ba1—Ba2—O10^i^	−25.53 (16)	O3—Ba1—O12—Ba2^iv^	−130.6 (3)
O1—Ba1—Ba2—N3	161.6 (4)	O14—Ba1—O12—Ba2^iv^	45.9 (2)
O11—Ba1—Ba2—N3	34.3 (3)	O10—Ba1—O12—Ba2^iv^	−9.8 (4)
O9—Ba1—Ba2—N3	−87.6 (3)	O13—Ba1—O12—Ba2^iv^	−77.0 (3)
O15—Ba1—Ba2—N3	−109.9 (3)	N1—Ba1—O12—Ba2^iv^	101.8 (3)
O3—Ba1—Ba2—N3	22.6 (4)	Ba2—Ba1—O12—Ba2^iv^	−167.9 (2)
O14—Ba1—Ba2—N3	119.2 (3)	O1—Ba1—O13—Ba2^iii^	−111.2 (3)
O10—Ba1—Ba2—N3	−47.5 (4)	O11—Ba1—O13—Ba2^iii^	121.5 (3)
O13—Ba1—Ba2—N3	−36.6 (3)	O9—Ba1—O13—Ba2^iii^	−95.3 (3)
O12—Ba1—Ba2—N3	83.2 (3)	O15—Ba1—O13—Ba2^iii^	−17.4 (2)
N1—Ba1—Ba2—N3	−175.0 (3)	O3—Ba1—O13—Ba2^iii^	−160.6 (3)
Ba2^iv^—Ba1—Ba2—N3	74.8 (2)	O14—Ba1—O13—Ba2^iii^	78.9 (3)
O1—Ba1—Ba2—Ba1^ii^	−93.2 (3)	O10—Ba1—O13—Ba2^iii^	47.0 (2)
O11—Ba1—Ba2—Ba1^ii^	139.50 (17)	O12—Ba1—O13—Ba2^iii^	151.9 (2)
O9—Ba1—Ba2—Ba1^ii^	17.6 (3)	N1—Ba1—O13—Ba2^iii^	−26.4 (5)
O15—Ba1—Ba2—Ba1^ii^	−4.63 (17)	Ba2—Ba1—O13—Ba2^iii^	−127.36 (19)
O3—Ba1—Ba2—Ba1^ii^	127.8 (3)	Ba2^iv^—Ba1—O13—Ba2^iii^	112.63 (19)
O14—Ba1—Ba2—Ba1^ii^	−135.59 (19)	O1—Ba1—O14—Ba2^iv^	−114.2 (3)
O10—Ba1—Ba2—Ba1^ii^	57.7 (4)	O11—Ba1—O14—Ba2^iv^	16.9 (2)
O13—Ba1—Ba2—Ba1^ii^	68.66 (16)	O9—Ba1—O14—Ba2^iv^	−135.3 (5)
O12—Ba1—Ba2—Ba1^ii^	−171.60 (17)	O15—Ba1—O14—Ba2^iv^	138.9 (2)
N1—Ba1—Ba2—Ba1^ii^	−69.8 (2)	O3—Ba1—O14—Ba2^iv^	−42.1 (4)
Ba2^iv^—Ba1—Ba2—Ba1^ii^	180.0	O10—Ba1—O14—Ba2^iv^	85.7 (3)
O1—Ba1—N1—C6	175.3 (12)	O13—Ba1—O14—Ba2^iv^	58.5 (4)
O11—Ba1—N1—C6	−70.4 (12)	O12—Ba1—O14—Ba2^iv^	−45.8 (2)
O9—Ba1—N1—C6	117.2 (11)	N1—Ba1—O14—Ba2^iv^	−165.7 (4)
O15—Ba1—N1—C6	45.6 (11)	Ba2—Ba1—O14—Ba2^iv^	−88.4 (2)
O3—Ba1—N1—C6	171.4 (10)	O1—Ba1—O15—Ba2^iii^	149.8 (3)
O14—Ba1—N1—C6	−74.5 (11)	O11—Ba1—O15—Ba2^iii^	−30.7 (4)
O10—Ba1—N1—C6	−1.7 (12)	O9—Ba1—O15—Ba2^iii^	93.2 (3)
O13—Ba1—N1—C6	55.0 (13)	O3—Ba1—O15—Ba2^iii^	69.9 (4)
O12—Ba1—N1—C6	−123.5 (11)	O14—Ba1—O15—Ba2^iii^	−111.3 (3)
Ba2—Ba1—N1—C6	157.4 (11)	O10—Ba1—O15—Ba2^iii^	−45.1 (2)
Ba2^iv^—Ba1—N1—C6	−83.8 (11)	O13—Ba1—O15—Ba2^iii^	17.9 (2)
O1—Ba1—N1—C2	−1.8 (8)	O12—Ba1—O15—Ba2^iii^	−126.1 (7)
O11—Ba1—N1—C2	112.5 (9)	N1—Ba1—O15—Ba2^iii^	−168.7 (4)
O9—Ba1—N1—C2	−59.9 (9)	Ba2—Ba1—O15—Ba2^iii^	108.1 (2)
O15—Ba1—N1—C2	−131.5 (9)	Ba2^iv^—Ba1—O15—Ba2^iii^	−77.5 (3)
O3—Ba1—N1—C2	−5.7 (10)	Ba1—O1—C1—O2	168.9 (9)
O14—Ba1—N1—C2	108.4 (9)	Ba2—O1—C1—O2	−36.7 (17)
O10—Ba1—N1—C2	−178.8 (9)	Ba1—O1—C1—C2	−11.6 (16)
O13—Ba1—N1—C2	−122.1 (8)	Ba2—O1—C1—C2	142.7 (9)
O12—Ba1—N1—C2	59.4 (9)	C6—N1—C2—C3	4(2)
Ba2—Ba1—N1—C2	−19.7 (9)	Ba1—N1—C2—C3	−178.6 (11)
Ba2^iv^—Ba1—N1—C2	99.1 (9)	C6—N1—C2—C1	−179.5 (12)
O3—Ba2—N3—C8	−2.1 (8)	Ba1—N1—C2—C1	−2.1 (14)
O15^i^—Ba2—N3—C8	112.3 (9)	O2—C1—C2—N1	−172.2 (12)
O11^ii^—Ba2—N3—C8	−130.5 (10)	O1—C1—C2—N1	8.3 (17)
O1—Ba2—N3—C8	−5.0 (11)	O2—C1—C2—C3	4.3 (19)
O9—Ba2—N3—C8	−59.5 (9)	O1—C1—C2—C3	−175.2 (13)
O13^i^—Ba2—N3—C8	110.3 (9)	C5—N2—C3—C2	3(2)
O12^ii^—Ba2—N3—C8	−178.2 (9)	N1—C2—C3—N2	−4(2)
O14^ii^—Ba2—N3—C8	−121.8 (9)	C1—C2—C3—N2	179.2 (13)
O10^i^—Ba2—N3—C8	60.2 (9)	C3—N2—C5—C6	−2(2)
Ba1—Ba2—N3—C8	−19.6 (9)	C2—N1—C6—C5	−3(2)
Ba1^ii^—Ba2—N3—C8	−137.5 (9)	Ba1—N1—C6—C5	180.0 (11)
O3—Ba2—N3—C12	175.7 (12)	N2—C5—C6—N1	2(3)
O15^i^—Ba2—N3—C12	−69.9 (12)	Ba2—O3—C7—O4	168.8 (10)
O11^ii^—Ba2—N3—C12	47.3 (11)	Ba1—O3—C7—O4	−34 (2)
O1—Ba2—N3—C12	172.8 (10)	Ba2—O3—C7—C8	−14.4 (18)
O9—Ba2—N3—C12	118.4 (11)	Ba1—O3—C7—C8	143.0 (9)
O13^i^—Ba2—N3—C12	−71.9 (11)	C12—N3—C8—C9	3.1 (19)
O12^ii^—Ba2—N3—C12	−0.3 (11)	Ba2—N3—C8—C9	−178.9 (10)
O14^ii^—Ba2—N3—C12	56.0 (12)	C12—N3—C8—C7	179.2 (12)
O10^i^—Ba2—N3—C12	−121.9 (11)	Ba2—N3—C8—C7	−2.7 (15)
Ba1—Ba2—N3—C12	158.2 (11)	O3—C7—C8—N3	10.2 (19)
Ba1^ii^—Ba2—N3—C12	40.4 (11)	O4—C7—C8—N3	−172.7 (12)
O11—Ba1—O1—C1	−125.4 (10)	O3—C7—C8—C9	−173.7 (13)
O9—Ba1—O1—C1	116.7 (11)	O4—C7—C8—C9	3(2)
O15—Ba1—O1—C1	54.1 (10)	C11—N4—C9—C8	1(2)
O3—Ba1—O1—C1	−175.8 (11)	N3—C8—C9—N4	−3(2)
O14—Ba1—O1—C1	−54.1 (10)	C7—C8—C9—N4	−179.0 (14)
O10—Ba1—O1—C1	15.7 (15)	C9—N4—C11—C12	1(2)
O13—Ba1—O1—C1	133.5 (9)	C8—N3—C12—C11	−1(2)
O12—Ba1—O1—C1	−109.5 (10)	Ba2—N3—C12—C11	−178.9 (11)
N1—Ba1—O1—C1	7.4 (9)	N4—C11—C12—N3	−1(3)
Ba2—Ba1—O1—C1	158.9 (12)	C18—N5—C14—C15	−1.2 (19)
Ba2^iv^—Ba1—O1—C1	−91.0 (10)	C18—N5—C14—C13	177.9 (13)
O11—Ba1—O1—Ba2	75.7 (4)	O5—C13—C14—N5	−0.9 (18)
O9—Ba1—O1—Ba2	−42.2 (3)	O6—C13—C14—N5	−178.8 (12)
O15—Ba1—O1—Ba2	−104.9 (3)	O5—C13—C14—C15	178.1 (12)
O3—Ba1—O1—Ba2	25.3 (3)	O6—C13—C14—C15	0.2 (19)
O14—Ba1—O1—Ba2	147.0 (3)	C17—N6—C15—C14	1(2)
O10—Ba1—O1—Ba2	−143.3 (6)	N5—C14—C15—N6	0(2)
O13—Ba1—O1—Ba2	−25.4 (5)	C13—C14—C15—N6	−178.9 (14)
O12—Ba1—O1—Ba2	91.5 (3)	C15—N6—C17—C18	−2(2)
N1—Ba1—O1—Ba2	−151.5 (4)	C14—N5—C18—C17	1(2)
Ba2^iv^—Ba1—O1—Ba2	110.1 (2)	N6—C17—C18—N5	1(3)
O3—Ba2—O1—C1	173.6 (10)	C24—N7—C20—C21	−2(2)
O15^i^—Ba2—O1—C1	30.4 (9)	C24—N7—C20—C19	178.0 (13)
O11^ii^—Ba2—O1—C1	−92.3 (10)	O8—C19—C20—N7	−173.2 (13)
O9—Ba2—O1—C1	−117.8 (10)	O7—C19—C20—N7	2.0 (19)
O13^i^—Ba2—O1—C1	88.1 (10)	O8—C19—C20—C21	7.2 (19)
O12^ii^—Ba2—O1—C1	−12.5 (11)	O7—C19—C20—C21	−177.6 (13)
O14^ii^—Ba2—O1—C1	−41.3 (9)	C23—N8—C21—C20	1(2)
O10^i^—Ba2—O1—C1	93.3 (10)	N7—C20—C21—N8	1(2)
N3—Ba2—O1—C1	176.1 (9)	C19—C20—C21—N8	−179.1 (14)
Ba1—Ba2—O1—C1	−160.1 (11)	C21—N8—C23—C24	−3(2)
Ba1^ii^—Ba2—O1—C1	−55.0 (10)	C20—N7—C24—C23	1(2)
O3—Ba2—O1—Ba1	−26.3 (3)	N8—C23—C24—N7	2(3)
O15^i^—Ba2—O1—Ba1	−169.5 (3)		

Symmetry codes: (i) *x*, *y*, *z*−1; (ii) *x*+1, *y*, *z*; (iii) *x*, *y*, *z*+1; (iv) *x*−1, *y*, *z*.

### Hydrogen-bond geometry (Å, °)

**Table:**

*D*—H···*A*	*D*—H	H···*A*	*D*···*A*	*D*—H···*A*
O10—H10A···O7	0.85	2.23	3.000 (13)	152
O10—H10B···O5	0.85	1.92	2.769 (11)	177
O11—H11A···O4	0.85	1.88	2.669 (12)	153
O11—H11B···O5	0.85	2.04	2.861 (12)	163
O11—H11B···N5	0.85	2.62	3.191 (14)	126
O13—H13A···N5	0.85	2.14	2.983 (14)	175
O14—H14A···O8	0.85	1.87	2.685 (12)	160
O9—H9A···O7^ii^	0.85	1.88	2.677 (12)	155
O9—H9B···O5^ii^	0.85	1.89	2.689 (12)	156
O13—H13B···O6^ii^	0.85	1.93	2.729 (12)	157
O15—H15A···O7^ii^	0.85	2.04	2.860 (12)	161
O15—H15A···N7^ii^	0.85	2.62	3.208 (14)	127
O12—H12A···O7^i^	0.85	1.96	2.811 (12)	179
O12—H12B···O5^i^	0.85	2.26	3.018 (12)	149
O14—H14B···N7^i^	0.85	2.18	3.028 (13)	174
O15—H15B···O2^iii^	0.85	1.90	2.676 (11)	152

Symmetry codes: (ii) *x*+1, *y*, *z*; (i) *x*, *y*, *z*−1; (iii) *x*, *y*, *z*+1.
